# Hypnosis can reduce pain in hospitalized older patients: a randomized controlled study

**DOI:** 10.1186/s12877-016-0180-y

**Published:** 2016-01-15

**Authors:** Sheila Ardigo, François R. Herrmann, Véronique Moret, Laurence Déramé, Sandra Giannelli, Gabriel Gold, Sophie Pautex

**Affiliations:** Division of Geriatrics, Geneva University Hospitals and Geneva University, Rue Gabrielle-Perret-Gentil 4, 1205 Genève, Switzerland; Division of Palliative Medicine, Geneva University Hospitals and Geneva University, Rue Gabrielle-Perret-Gentil 4, 1205 Genève, Switzerland; Division of Primary Care, Geneva University Hospitals and Geneva University, Rue Gabrielle-Perret-Gentil 4, 1205 Genève, Switzerland

**Keywords:** Hypnosis, chronic pain, older patients

## Abstract

**Background:**

Chronic pain is a common and serious health problem in older patients. Treatment often includes non pharmacological approaches despite a relatively modest evidence base in this population. Hypnosis has been used in younger adults with positive results. The main objective of this study was to measure the feasibility and efficacy of hypnosis (including self hypnosis) in the management of chronic pain in older hospitalized patients.

**Methods:**

A single center randomized controlled trial using a two arm parallel group design (hypnosis versus massage). Inclusion criteria were chronic pain for more than 3 months with impact on daily life activities, intensity of > 4; adapted analgesic treatment; no cognitive impairment. Brief pain inventory was completed.

**Results:**

Fifty-three patients were included (mean age: 80.6 ± 8.2- 14 men; 26 hypnosis; 27 massage. Pain intensity decreased significantly in both groups after each session. Average pain measured by the brief pain index sustained a greater decrease in the hypnosis group compared to the massage group during the hospitalisation. This was confirmed by the measure of intensity of the pain before each session that decreased only in the hypnosis group over time (P = 0.008). Depression scores improved significantly over the time only in the hypnosis group (P = 0.049). There was no effect in either group 3 months post hospitals discharge.

**Discussions and conclusion:**

Hypnosis represents a safe and valuable tool in chronic pain management of hospitalized older patients. In hospital interventions did not provide long term post discharge relief.

**Trial registration:**

ISRCTN15615614; registered 2/1/2015.

## Background

Chronic pain is a common and serious health problem in the elderly. Various epidemiological studies estimate the prevalence between 25 and 65% in elderly persons who live in the community and up to 80% in institutionalized elderly people [1–3]. The most common chronic, non-malignant pain conditions in the elderly are musculoskeletal pain located in the joints and back due to osteoarthritis or osteoporosis fractures in addition to neuropathic pain such as post-herpetic neuralgia and peripheral neuropathy [4]. Living with pain has an impact on elderly people’s overall functioning and quality of life. Patients suffering from chronic pain often have depression, sleep disturbances and impaired functionality with an impact on their quality of life [5].

The management of an elderly patient with chronic pain includes both pharmacological and non-pharmacological treatment. Common drug classes are paracetamol, nonsteroidal antiinflammatory drugs (NSAIDs), and opioids [6]. The increased risk of polypharmacy, adverse side-effects and intoxication in elderly adults, compared to younger adults, is well recognized, leading to an increased interest in non pharmacological approaches [7]. These include psychological support, physiotherapy, massage or hypnosis for example.

Hypnosis is an altered state of consciousness or state of focused attention to verbal stimuli induced by the therapist (hetero-hypnosis) or the subject himself (self-hypnosis) [8, 9]. To enter “hypnosis” means “to enter” in “another” state, a transition from a normal ordinary state of consciousness [10]. The patient is always in control and can stop the process whenever he desires to do so. Hypnotic techniques have proven to be useful for different kind of pain especially pain associated with burns, cancer, invasive medical procedures, headaches, musculoskeletal conditions, irritable bowel syndrome, and fibromyalgia for example [11–14]. The practice of self-hypnosis has been shown in studies to be an important element in the long term control of chronic pain [15]. Self-hypnosis can be taught to the patient as a tool to modify behavior regarding nociceptive perception. It allows him to take an active part in his own pain management using personal resources and experiences. Little research specifically addresses the use of hypnosis in older adults. Although three studies did include older patients, [16–18]. The mean age of participants was relatively young (60 to 69 years) and not representative of very old patients treated in geriatric settings.

The main objective of this randomized controlled study was to measure how hypnosis can improve the management of chronic pain in older hospitalized patients. Our hypothesis was that hypnosis would be feasible and effective in decreasing pain intensity in this population. We further hypothesized that the ability to perform self hypnosis would provide a prolonged decrease in pain intensity measured 3 months after hospital discharge compared to massage.

## Methods

### Design of the study

A single center randomized controlled trial using a two arm design (hypnosis versus massage) to assess the immediate and prolonged effect of hypnosis on the management of chronic pain in elderly patients. The study setting was a large geriatric hospital (300 beds).

### Participants

Potentially eligible patients were identified by the team in charge of the patient or by the pain and palliative care consultation team. Patients were included starting from the fifth day of hospitalization, after stabilization of their acute illnesses.

Inclusion criteria were chronic pain for more than 3 months with impact on daily living activities. Intensity of pain had to be higher than 4 on a numerical pain rating scale (0–10) at inclusion despite adequate analgesia. Exclusion Criteria were: deafness; patient in his last days of life, psychosis, delirium (according to DSM-IV); cognitive impairment (Mini mental Status examination >25) [19] post-traumatic stress disease; active skin disease with a contraindication for massage.

### Outcomes

#### Primary outcome

The third question of the Brief Pain Inventory (BPI) (average pain) measured at inclusion (T0), week one (T1) and two (T2), at discharge (T3) and 12 weeks (T12) later was used. This item is rated on a 0 to 10 scale, where 0 = no pain and 10 = pain as bad you can imagine. The rater who completed the BPI was blinded to the allocation group [20].

#### Secondary outcome

The second part (pain interference with daily activities; 7 items) of the BPI measured at inclusion, at T0, T1, T2 and T3 [20]. The BPI measures interference of pain with daily activities over the last 24 hours (mood, walking ability, normal work [including household], relationships, sleep, and enjoyment of life).The items are rated on a 0 to 10 scale, where 0 = no interference and 10 = interference as bad you can imagine. Mean interference score was calculated [21, 22].

The patient assessed his intensity of pain before and after the sessions of hypnosis and massage with a numerical rating scale rating from 0 (no pain) to 10 (pain as bad as you could imagine).

Analgesic use at T0, T1, T2 and T3 was collected and doses were converted to the morphine equivalent in milligrams.

To assess anxiety and mood, we used the Hospital Anxiety and Depression Scale (HADS) [23]. It consists of 14 questions, seven for anxiety and seven for depression. Each item was answered by the patient on a four point (0–3) response category so the possible scores ranged from 0 to 21 for anxiety and 0 to 21 for depression. The HADS was completed at T0, T1, T2 and T3.

The functional performance of the patients was assessed by the Functional Independence Measure (FIM) completed by the team in charge of the patient at T0, T1, T2 and T3 [24].

### Collected data

Baseline demographic data (age, gender, education, marital status), as well as primary diagnosis and the co-morbidities measured by the Cumulative Illness Rating Scale-Geriatrics were collected [25]. Detailed characteristics of pain were collected as well as French versions of the McGill Pain Questionnaire (MPQ). This instrument describes sensory, affective and mixed sensory aspects of pain [26]. Previous and actual specific treatments were collected.

### Randomization

A randomization list to attribute patients to the two treatment groups was obtained with Stata “ralloc” function with an allocation ratio of 1, randomized block sizes ranging from 4 to 8 and no stratification.

To guarantee allocation concealment the clinician called one of the authors who was not in charge of the patient and was responsible for randomized allocation.

### Ethics approval

All procedures performed in studies involving human participants were in accordance with the ethical standards of the institutional and/or national research committee and with the 1964 Helsinki declaration and its later amendments or comparable ethical standards. The Ethics Commission of the University Hospital Geneva approved the study. Written informed consent was signed by each participant.

### Intervention

#### Hypnosis

The session was designed as suggested by Jensen and Petterson as a “brief hypnosis treatment” [14]. Three sessions of 30 minutes (once a week according to the general condition of the patient) were conducted by a physician trained in medical hypnosis. We opted for a short number of sessions, because of the patients’ length of hospitalization. Before the session it was explained to the patients that the intervention consists in teaching them specific skills to help provide pain relief. The session was divided in the classical phases of hypnosis including induction, deepening and post hypnotic suggestions. During induction, patients were asked to imagine themselves in a nice place and to make some suggestions (selected according to their personal history) for analgesia and comfort. We practiced deepening and post hypnotic suggestions to obtain an effect of the treatment on the long run and to encourage the practice of self-hypnosis. Post-hypnotic suggestions were given by the therapist during the session that allow anchor and influence in the therapeutic goal established with the patient's perception of pain, time, memory, anxiety. Self-hypnosis was taught to the patient, with the aim to give them some form of control over pain.

#### Massages

Massage is a technique that provides relaxation and improves well-being which helps reduce the feeling of pain [27, 28]. Three sessions of 30 minutes (once a week according to the general condition of the patient) were conducted by a nurse with a certification in massage. Patient was comfortably installed in a quiet room. At each session the patient could choose the area of massage: back massage or hands and arms or legs and feet (possibly abdomen or face).

#### Power calculation

The size of the group was determined from the results of a randomized controlled study comparing self-hypnosis and relaxation in patients with chronic pain in the setting of patients with chronic pain secondary to multiple sclerosis [29]. A power of 95% to detect a difference of 2 points on the average pain scale) was used.

#### Statistical analysis

Descriptive statistics were reported as mean ± standard deviation for parametric data, median and inter-quartile range for non-parametric data. The hypnosis- and massage-groups were compared at baseline using t-tests or Fisher’s exact test as appropriate. Longitudinal data were analyzed according to the intention-to-treat concept (without data imputation) using linear mixed-effects regression models taking into account random effects (participant) and fixed effects (hypnosis vs massage) to predict the primary end-point with time-points, hypnosis and their interaction term as exploratory variables. These models take the repeated measure design of the study into account and allow for a different number of observations within subjects. They yield unbiased estimates of the effect size of the intervention (difference between hypnosis and massage) under the assumption that values are missing at random. Effect sizes are reported in the results section for all comparisons that showed significant between group differences over time. The level of significance was set at p < 0.05.

Analyses were performed using Stata 12.1 (STATA Corporation, College Station, Tx, USA).

## Results

**Fig. 1:**
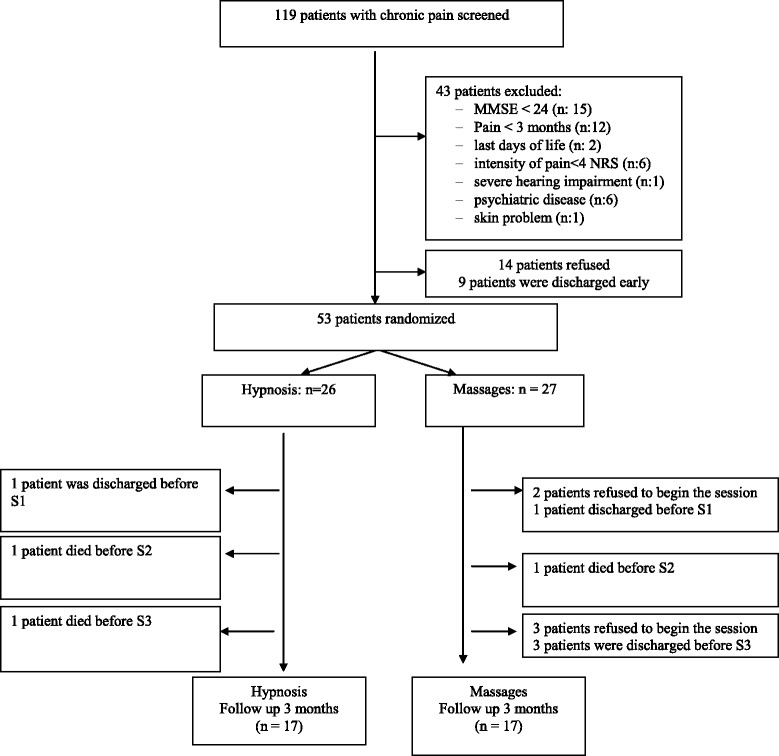
Flow-chart

Over a 12-month period, 119 charts were screened (Fig. 1). A total of 53 patients were finally included in the study. They were included 13 days (median) after admission. Main characteristics of the patients are described in Table 1. There was no significant difference between both groups (P > 0.05), except that more patients were hospitalized because of their pain condition in the hypnosis group. All patients were living at home before admission to hospital.

**Table 1:** Baseline characteristics of the patients

		Hypnosis	Massage	Total
		n:26	n:27	n:53
Age (mean ± SD)		81.4 ± 1.5	79.9 ± 8.5	80.6 ± 8.2
Gender M/F		5/21	9/18	14/39
Education n (%)				
	Compulsory school	8 (31)	5 (19)	13 (25)
	Diploma	10 (38)	17 (62)	27 (50)
	University	8 (31)	5 (19)	13 (25)
Marital status n (%)				
	alone	2 (12)	5 (19)	8 (15)
	married	9 (35)	11 (41)	20 (38)
	widowed	9 (35)	9 (33)	18 (34)
	divorced	5 (18)	2(7)	7 (13)
Admission reason n (%)				
	Pain condition*	20 (73)	14 (52)	34 (64)
	cardiac	1 (4)	1 (4)	2 (4)
	infection	2 (8)	1 (4)	3 (6)
	fall	2 (8)	5 (18)	7 (13)
	fracture	0	2 (8)	2 (4)
	other	1 (4)	4 (14)	5 (9)
MMSE (mean ± SD)		26.7 ± 2.0	27.1 ± 1.9	26.9 ± 2.1
CIRS (mean ± SD)		13.8 ± 4.8	14.7 ± 4.2	13.9 ± 4.5
HADS anxiety (n)	score			
	0-7	17	19	36
	8-11	4	3	7
	>11	5	5	10
HADS depression (n)	0-7	22	23	55
	8-11	2	2	4
	11	2	2	4

*P = 0.021

Pain characteristics: pain etiologies (n;%) were chronic back pain (26; 49%), neuropathic pain (8; 16%), osteoarthritis (knees (3;6%) ankle (4; 8%); shoulder (4; 8%)), fibromyalgia (5; 9%); and other (3; 6%). Pain lasted for 6.3 ± 4.2 years. Prescribed drugs (n;%) before admission were paracetamol (acetaminophen) (45; 85%) opioids (principally morphine) (40; 75%), and NSAIDS (15; 28%), antidepressant (15; 28%) and antiepileptics (29; 55%). The only statistical significant difference regarding the characteristics of pain between both groups was the number of patients with osteoarthritis of the shoulder (0 in the hypnosis group; 4 in the massage group; P = 0.04). On the French version of the McGill Pain Questionnaire (MPQ) descriptors of pain consist mostly in affects: two words were chosen by more than one-third of the patients: tender (36%) and ‘annoying’ (41%).

Detailed results of the BPI are described in Table 2. Pain was located mainly in the back and lower limbs. Average pain intensity was rather high in this population and the interference of pain was important on general activity, walking and household activities. The impact of pain on sleep or on social relations was less important.

**Table 2:** Brief Pain Inventory

		Hypnosis	Massage	Total	P
		n:26	n:27	n:53	
Severity	Worst pain	7.6 ± 2.1	7.2 ± 2.6	7.4 ± 2.1	0.58
	Least pain	3.5 ± 2.8	3.2 ± 2.4	3.4 ± 2.6	0.70
	Pain on average	5.6 ± 2.4	5.3 ± 1.9	5.5 ± 2.2	0.58
	Current pain	4.7 ± 3.2	4.5 ± 3.1	4.5 ± 3.1	0.51
Interference	General activity	6.0 ± 3.2	6.0 ± 3.5	6.0 ± 3.4	0.93
	Mood	3.4 ± 3.4	3.6 ± 3.4	3.5 ± 3.3	0.81
	Walking ability	5.6 ± 3.8	5.4 ± 4.0	5.5 ± 3.8	0.87
	Normal work (household)	6.0 ± 3.3	5.7 ± 3.7	5.8 ± 3.5	0.62
	Relationships with people	3.1 ± 3.5	2.6 ± 3.1	2.9 ± 3.3	0.51
	Sleep	3.6 ± 3.8	2.4 ± 3.0	2.8 ± 3.5	0.11
	Enjoyment of life	3.2 ± 3.6	2.5 ± 3.1	2.9 ± 3.3	0.45
	Mean interference score	3.9 ± 2.0	4.3 ± 2.7	4.2 ± 2.4	0.52

Prescribed drugs (n;%) at inclusion were paracetamol (acetaminophen) (42; 79%) opioids (principally morphine) (35; 66%), and NSAIDS (17; 32%), antidepressant (12; 23%) and antiepileptics (31; 58%). There were no statistically significant differences between both groups. Adjusting all models for quantity of morphine equivalent in mg did not modify the observed results. Quantity of morphine equivalent was never found significant in any regression models.Hypnosis and massage feasibility:

Twenty-three patients (88% of total in group) completed the 3 sessions of hypnosis and 17 (63% of total in group) the 3 sessions of massage. Reasons to stop the sessions were the patient died (n:3), early discharge (n:5) and patients’ refusal (n:5).

**Fig. 2:**
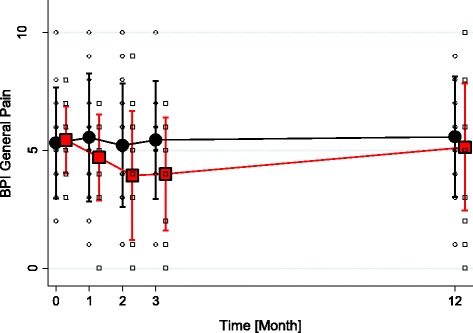
Evolution of Brief pain inventory: Pain on average (0–10) (red square: hypnosis; black circle: massage)

Changes between sessions in the average pain measured by the BPI: (Table 3; Fig. 2) there was a non significant decrease of mean pain intensity in the hypnosis group over time (T0, T1, T2, T3) compared to the massage group: T0 vs T2, P = 0.041 ;T0 vs T3, P = 0.071. There was no effect at T12.

**Table 3:** Assessment of patients at inclusion, week 1, week 2, week 3 and week 12

	T0		T1		T2		T3		T12	
	Hypnosis	Massage	Hypnosis	Massage	Hypnosis	Massage	Hypnosis	Massage	Hypnosis	Massage
	N: 26	N:27	N: 25	N:24	N: 24	N:21	N: 23	N:17	N: 17	N:17
BPI Pain on average (0–10)	5.4 ± 1.4	5.5 ± 2.5	4.7 ± 1.8	5.6 ± 2.7	4.1 ± 2.8	5.2 ± 2.6	4.0 ± 2.4	5.4 ± 2.5	4.6 ± 5.0	5.4 ± 2.5
BPI pain interference score (0–10)	3.9 ± 2.0	4.3 ± 2.7	3.9 ± 1.9	3.1 ± 2.2	3.1 ± 2.8	3.3 ± 2.3	2.0 ± 2.0	2.3 ± 2.0±	3.0 ± 2.8	3.6 ± 2.1
Pain (0–10) before session			5.8 ± 2.4	4.8 ± 2.7	4.5 ± 2.2	5.3 ± 2.9	3.8 ± 2.3	5.8 ± 2.6		
Pain (0–10) after session			3.8 ± 2.6	2.5 ± 2.9	3.4 ± 2.3	2.7 ± 2.4	2.6 ± 2.8	3.6 ± 2.6		
HADS anxiety (0–18)	4.7 ± 4.6	4.5 ± 4.3	4.1 ± 4.5	4.4 ± 4.1	3.4 ± 2.9	3.9 ± 3.7	3.8 ± 4.6	3.4 ± 2.9		
HADS depression (0–18)	4.5 ± 2.0	4.9 ± 3.7	3.6 ± 1.8	4.9 ± 4.0	3.3 ± 2.3	6.1 ± 4.0	2.8 ± 2.2	4.6 ± 3.5		
FIM score (0–117)	90.1 ± 25.0	94.3 ± 22.1	95.3 ± 24.0	89.6 ± 24.6	92.6 ± 27.6	94.9 ± 27.9	101.3 ± 24.8	100.8 ± 24.4		
Morphine mg	31.8 ± 39.0	20.7 ± 41.5	34.5 ± 40.0	25.2 ± 34.0	19.7 ± 29.9	21.3 ± 29.4	27.6 ± 37.9	21.0 ± 38.7

Changes between sessions in the pain interference score: the score decreased in both groups over time: T0 vs T1, P = 0.021; T0 vs T2, P = 0.07; T0 vs T3; P = 0.009 T0 vs T12, P:0.075). There was no difference between both groups (hypnosis compared to massage).

Changes in intensity of pain before and after the session: Both groups demonstrated a statistically significant decrease in pain intensity after the sessions (P = 0.041- P = 0.034 respectively for the hypnosis and massage group).

**Fig. 3:**
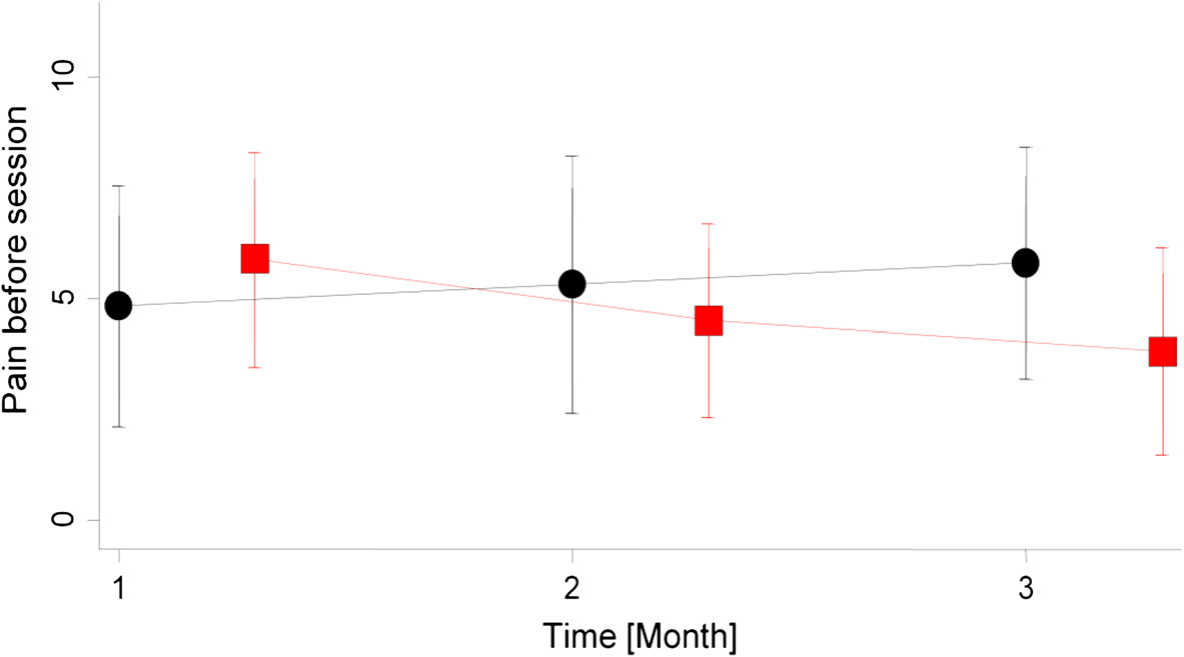
Evolution of Pain (0–10) at baseline and before each session (0–10) red square: hypnosis; black circle: massage)

There was a significant decrease of the intensity of the pain before the session in the hypnosis group over time (T1, T2, T3) compared to the massage group: (T1 vs T2, P = 0.032 ;T1 vs T3, P = 0.008) (Fig. 3). The adjusted effect sizes (difference between intervention and massage effects on the 10 point BPI intensity scale) were (T1 vs T2, −2.25 P = 0.029 ;T1 vs T3, −3.13 P = 0.011).

Changes in the score of HADS: Scores of depression improved significantly over the time in the hypnosis group (significant time effect T1 vs T2; P = 0.049). There was no effect on anxiety.

Changes in the functional status (FIM) of the patients and the quantity of morphine equivalent in mg: we could not demonstrate a significant group effect (hypnosis compared to massage), or time effect (between T0, T1, T2 and T3), or time*treatment interaction.

Patients were discharged on average at 19.2 ± 4.4 days after inclusion. One patient died during the follow-up period. No patients had unexpected feelings, thoughts or behavior after or during the hypnotic treatment. Only three patients used auto-hypnosis after discharge (one patient 1x/week); two patients 3/week.

Previous results from mixed regression models were unchanged after adjusting for pain as a reason for hospitalization, which differs between the groups after randomization."

## Discussion

The results of this study provide interesting information about hypnosis in the management of pain in very old hospitalized patients.

First, we demonstrated that hypnosis was feasible in a hospitalized older population and that chronic pain significantly decreased after the sessions. Second we confirmed our hypothesis that hypnosis had a more prolonged analgesic effect compared to massage throughout the hospital stay. Although little research addresses this issue in this population our results are supported by Ashton who found that patients who were taught self-hypnosis before coronary artery bypass surgery (mean age 64 years) needed less postoperative pain medication and had less postoperative anxiety and tension compared to a control group [16–18]. In another study comparing hypnosis and relaxation, Gay reported that hypnosis reduced the amount of analgesic medication needed to control arthritic pain in older adults with osteoarthritis (mean age 64 years) [18]. Lang reported that older adults (mean age 69 years) used less analgesics, and reported less pain when self-hypnotic relaxation was used during interventional radiologic procedures [17].

Third, hypnosis had a positive effect on mood; this was not the case for massage. Possibly, hypnosis may allow patients to refocus on their abilities and resources leading to better control of their own symptoms. Post-hypnotic suggestions may also play a role. Fourth, hypnosis resulted in no adverse effects confirming its safety in older people.

Contrary to our hypothesis, we found no difference in pain intensity and mood between the hypnosis and massage groups at 12 weeks post discharge. This is most likely related to the very low number of patients that continued to practice self-hypnosis three months after their discharge from hospital and suggests that further attempts at long term pain control through hypnosis and self-hypnosis should include post-discharge interventions such as reminders and efforts to maintain motivation. Audio-tapes of the sessions may also prove valuable in this regard as they encourage the practice of self-hypnosis [30].

Recruitment was difficult and the failure to obtain differences across groups on pain interference and anxiety scores may be related to lack of power. Another limitation is the short duration of the intervention. Most studies including younger patients with chronic pain used at least 6 sessions of hypnosis [31–36]. Jensens recommends 4 to 7 sessions for “brief hypnosis treatment” and eight and more sessions for a full hypnosis treatment. This number of sessions is difficult to obtain in an older population with a many functional limitations, especially in the context of current pressures to shorten lengths of stay.

## Conclusion

Despite the above limitations, our findings demonstrate that self-hypnosis is safe and feasible in very old hospitalized patients with multiples co-morbidities. Furthermore this technique reduced pain intensity and had a positive effect on mood. Our results indicate that hypnosis and self-hypnosis may be valuable tools in chronic pain management and suggest that health providers caring for older patients with chronic pain should be trained in this treatment modality so that it could be applied in conjunction with pharmacological treatment.

Health care provider should be aware that this treatment can be provided safely and efficiently by trained physicians and nurses.

## References

[1] Helme RD, Gibson SJ (2001). The epidemiology of pain in elderly people. Clin Geriatr Med.

[2] Blyth FM, March LM, Brnabic AJ, Jorm LR, Williamson M, Cousins MJ (2001). Chronic pain in Australia: a prevalence study. Pain.

[3] Ferrell BA (1995). Pain evaluation and management in the nursing home. Ann Intern Med.

[4] The management of persistent pain in older persons. J Am Geriatr Soc, 2002. 50: S205-24. 10.1046/j.1532-5415.50.6s.1.x 12067390

[5] Ashburn MA, Staats PS (1999). Management of chronic pain. Lancet.

[6] Holt PR, Kozuch P, Mewar S (2009). Colon cancer and the elderly: from screening to treatment in management of GI disease in the elderly. Best Pract Res Clin Gastroenterol.

[7] Hanlon JT, Guay DRP, Ives TJ. Oral analgesics: Efficacy moa, pharmacokinetics, adverse, effects di, and practical recommendations for, use in older adults. In: Gibson SJ, Weiner DK (Eds.) P management iopPipra, Vol. 35 pSIP. 2005.

[8] Maquet P (1999). Brain mechanisms of sleep: contribution of neuroimaging techniques. J Psychopharmacol.

[9] Silbersweig DA, Stern E, Frith C, Cahill C, Holmes A, Grootoonk S (1995). A functional neuroanatomy of hallucinations in schizophrenia. Nature.

[10] Hrezo RJ (1998). Hypnosis: an alternative in pain management for nurse practitioners. Nurse Pract Forum.

[11] Haanen HC, Hoenderdos HT, van Romunde LK, Hop WC, Mallee C, Terwiel JP (1991). Controlled trial of hypnotherapy in the treatment of refractory fibromyalgia. J Rheumatol.

[12] Lynch DF (1999). Empowering the patient: hypnosis in the management of cancer, surgical disease and chronic pain. Am J Clin Hypn.

[13] Montgomery GH, DuHamel KN, Redd WH (2000). A meta-analysis of hypnotically induced analgesia: how effective is hypnosis?. Int J Clin Exp Hypn.

[14] Elkins G, Jensen MP, Patterson DR (2007). Hypnotherapy for the management of chronic pain. Int J Clin Exp Hypn.

[15] Jensen M, Patterson DR (2006). Hypnotic treatment of chronic pain. J Behav Med.

[16] Ashton C, Whitworth GC, Seldomridge JA, Shapiro PA, Weinberg AD, Michler RE (1997). Self-hypnosis reduces anxiety following coronary artery bypass surgery. A prospective, randomized trial. J Cardiovasc Surg (Torino).

[17] Lang EV, Joyce JS, Spiegel D, Hamilton D, Lee KK (1996). Self-hypnotic relaxation during interventional radiological procedures: effects on pain perception and intravenous drug use. Int J Clin Exp Hypn.

[18] Gay MC, Philippot P, Luminet O (2002). Differential effectiveness of psychological interventions for reducing osteoarthritis pain: a comparison of Erikson [correction of Erickson] hypnosis and Jacobson relaxation. Eur J Pain.

[19] Folstein MF, Folstein SE, McHugh PR (1975). "Mini-mental state". A practical method for grading the cognitive state of patients for the clinician. J Psychiatr Res.

[20] Poundja J, Fikretoglu D, Guay S, Brunet A (2007). Validation of the French version of the brief pain inventory in Canadian veterans suffering from traumatic stress. J Pain Symptom Manage.

[21] Tsui JI, Herman DS, Kettavong M, Anderson BJ, Stein MD (2011). Escitalopram is associated with reductions in pain severity and pain interference in opioid dependent patients with depressive symptoms. Pain.

[22] Cleeland CS, Ryan KM (1994). Pain assessment: global use of the Brief Pain Inventory. Ann Acad Med Singapore.

[23] Zigmond AS, Snaith RP (1983). The hospital anxiety and depression scale. Acta Psychiatr Scand.

[24] Linacre JM, Heinemann AW, Wright BD, Granger CV, Hamilton BB (1994). The structure and stability of the Functional Independence Measure. Arch Phys Med Rehabil.

[25] Zekry D, Valle BH, Michel JP, Esposito F, Gold G, Krause KH, et al. Prospective comparison of six co-morbidity indices as predictors of 5 years post hospital discharge survival in the elderly. Rejuvenation Res. 2010;13(6):675–682. 10.1089/rej.2010.1037 20818930

[26] Boureau F, Luu M, Doubrere JF (1992). Comparative study of the validity of four French McGill Pain Questionnaire (MPQ) versions. Pain.

[27] Wilkinson S, Aldridge J, Salmon I, Cain E, Wilson B (1999). An evaluation of aromatherapy massage in palliative care. Palliat Med.

[28] Kutner JS, Smith MC, Corbin L, Hemphill L, Benton K, Mellis BK (2008). Massage therapy versus simple touch to improve pain and mood in patients with advanced cancer: a randomized trial. Ann Intern Med.

[29] Jensen MP, Hanley MA, Engel JM, Romano JM, Barber J, Cardenas DD (2005). Hypnotic analgesia for chronic pain in persons with disabilities: a case series. Int J Clin Exp Hypn.

[30] Jensen MP, Hanley MA, Engel JM, Romano JM, Barber J, Cardenas DD (2005). Hypnotic analgesia for chronic pain in persons with disabilities: a case series. Int J Clin Exp Hypn.

[31] Spiegel D, Bloom JR (1983). Group therapy and hypnosis reduce metastatic breast carcinoma pain. Psychosom Med.

[32] Picard P, Jusseaume C, Boutet M, Duale C, Mulliez A, Aublet-Cuvellier B (2013). Hypnosis for management of fibromyalgia. Int J Clin Exp Hypn.

[33] Castel A, Cascon R, Padrol A, Sala J, Rull M (2012). Multicomponent cognitive-behavioral group therapy with hypnosis for the treatment of fibromyalgia: long-term outcome. J Pain.

[34] Bernardy K, Fuber N, Klose P, Hauser W (2011). Efficacy of hypnosis/guided imagery in fibromyalgia syndrome--a systematic review and meta-analysis of controlled trials. BMC Musculoskelet Disord.

[35] Melzack R, Perry C (1975). Self-regulation of pain: the use of alpha-feedback and hypnotic training for the control of chronic pain. Exp Neurol.

[36] Edelson J, Fitzpatrick JL (1989). A comparison of cognitive-behavioral and hypnotic treatments of chronic pain. J Clin Psychol.

